# Gut Microbiome Influences the Efficacy of Neuropsychiatric Drugs

**DOI:** 10.34172/apb.47093

**Published:** 2026-06-01

**Authors:** Anjal Mohamed Ageel, Kannan Subbaram, Sheeza Ali, Queen Alice Arul

**Affiliations:** ^1^School of Medicine, The Maldives National University, Male’, Maldives; ^2^All India Institute of Medical Sciences, Kalyani, India

## To Editor,

 The gut microbiome is a complex ecosystem of commensal, symbiotic, and pathogenic microorganisms, comprising approximately 3.8 × 10¹³ bacteria, along with viruses, fungi, and archaea, whose collective genes outnumber those of the host by more than 100-fold. These organisms have their roles in keeping the host healthy. ^1,2^ Over 95% of gut microbiota includes *Enterobacteria, Proteobacteria, Fusobacteria, Actinobacteria, Verrucomicrobia, Bacteroidetes, Firmicutes*.^3^ *Lactobacillus, Bacillus, Enterococcus, Clostridium* and *Ruminococcus*, are among the more than 200 genera that make up the *Firmicutes*. *Bacteroidetes* mainly include *Bacteroides* and *Prevotella*, whereas *Actinobacteria*, though less abundant, is largely represented by *Bifidobacterium* in the gut.^3,4^

 Given the complexity and importance of the gut microbiome, perturbations in the gut microbiome (dysbiosis) can profoundly affect the host’s health. Oral medications can induce dysbiosis, which may act as both a cause and consequence of various disorders. Dysbiosis reduces the quantity of good bacteria, leading to poor production of energy, bile acid, carbohydrate fermentation, and other important metabolic functions that take place because of this.

 The gut microbiome significantly affects the metabolism and bioavailability of orally taken neuropsychiatric drugs.

 The gut microbiota exerts both direct and indirect impacts on the drug metabolism through enzymatic reactions. It includes deacetylation, demethylation, and de-hydroxylation of an orally taken drug before the drug becomes bioavailable. Hence, the gut microbiome has a profound effect on drug breakdown, efficiency, and their toxicity^5^

 Some bacteria may accumulate drugs or affect the gene expression of the drug transporters in the host cells, including GPI protein and cytochrome-P450, which affect the bioavailability and pharmacokinetic properties of the drugs.^5^ Moreover, the psychotropic drugs affect the composition of the microbiota, which later affects the drug efficacy and bioavailability of drugs used subsequently.^6^ There is also evidence emerging that the variability in drug response among individuals is also attributable to the variability in the composition of the microbiota of the human gut, aside from the classical pharmacogenomic principles.^7^ However, it is important to note that most of the evidence is from in vitro or animal studies, and so data pertaining to humans is very limited.

 It has come to light that the gut microbiome plays a crucial role in psychiatric illness. A systematic review found that depleted levels of *Faecalibacterium*and *Coprococcus*and enriched levels of *Eggerthella*were consistently shared between major depressive disorder, bipolar disorder, psychosis and schizophrenia, and anxiety. This study further supports that these psychiatric conditions are linked with reduced levels of butyrate-producing anti-inflammatory bacteria and increased levels of bacteria that are pro-inflammatory.^8^ As short-chain fatty acid (SCFA) levels reduce due to reduced butyrate producing bacteria, it increases systemic and CNS inflammation and disrupts neurotransmitter balance, contributing to depressive symptoms, mood swings, and cognitive disturbances. Other bacteria, *Atopobium* and *Veillonella* can significantly increase the inflammatory markers that affect cognitive and neurocircuitry function in schizophrenia patients. *Eggerthella* spp. are observed to be associated with enhanced inflammation in bipolar disorder and mood swing patients.^8^

 Emerging evidence suggests that through its impact on drug metabolism, intestinal uptake, and host physiological responses, the alterations in the gut microbiota may have a substantial impact on the efficacy of drugs prescribed for psychiatric purposes. These associations are influenced by disease states, medications taken together, diet, and environmental factors. The gut bacteria can metabolically inactivate the active drugs into inactive metabolites and reduce systemic bioavailability, hence reducing the therapeutic response.^9^ For example, *Enterococcus faecalis *expresses tyrosine decarboxylase, which transforms L-DOPA into dopamine in the lumen, thus impeding optimal delivery of the drug to the brain. This process is further enhanced by *Eggerthella lenta*, which transforms dopamine into m-tyramine through a molybdenum-dependent dehydroxylase and results in additional loss of the active compound (Figure 1).

**Figure 1:**
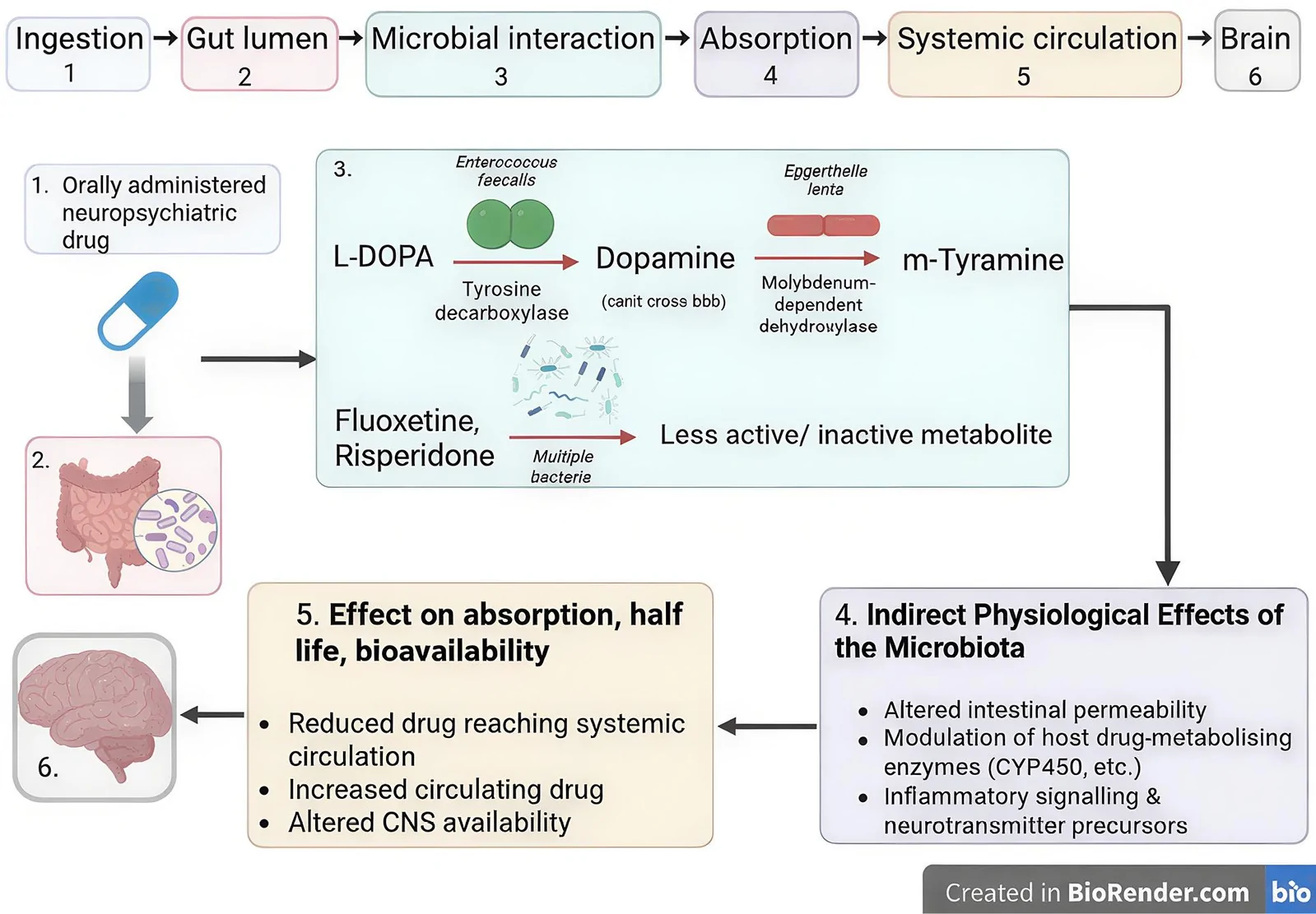
Influence of gut microbiome on the efficacy of psychotropic drugs. The figure illustrates how the gut microbiota influences the neuropsychiatric drugs from ingestion to brain effects. Microbial enzymes in the gut can directly metabolize drugs (e.g.: converting L-DOPA to dopamine and further to inactive metabolites), reducing the drugs bioavailability. Additionally, the microbiota indirectly alters the drug absorption, metabolism (e.g.: CYP450 activity), and intestinal permeability, ultimately affecting the systemic drug levels half-life, and central nervous system availability

 Microbial transformation has also been reported for other psychotropic agents. In vitro, studies revealed that different strains of gut bacteria metabolize fluoxetine, and microbial reductases are also capable of modifying the benzoxazole ring of risperidone, which may enhance its elimination.^6,9^ All these findings collectively depict the way microbial enzymatic processes like decarboxylation, dehydroxylation, reduction, acetylation, and hydrolysis diminish the potency of the drug or curtail the effective half-life of psychiatric medications by reduced systemic drug exposure.^7,9^ The altered composition of gut microbiota in psychiatric disorders further underlines the potential clinical relevance of these drug-microbiota interactions.^8^

 Simultaneously, gut microbes may also enhance drug activity under certain conditions, which illustrates a bidirectional influence on pharmacotherapy. Microbes possessing β-glucuronidases and sulfates can deconjugate drug metabolites and thereby re-release active parent compounds into the host’s gut lumen, enabling re-absorption and enterohepatic circulation, which can increase the drug’s half-life and exposure.^9^ Moreover, microbiota can exert indirect effects on the drug’s fate by modulating the intestinal barrier function, influencing host expression of phase I and phase II drug-metabolizing enzymes and transporters, and altering the local inflammatory or metabolic environment so that it will affect how much drug is being absorbed, distributed, or cleared by the body.^9^ In combination, these microbial direct and indirect mechanisms create a substantial interindividual variability in the drug effects, which helps to explain why two patients receiving the same medication may respond very differently depending on their gut microbial composition.

 To counteract this, there are several methods and strategies that could be employed. First, identifying patients with dysbiosis before or during treatment can help to anticipate altered drug metabolism or bioavailability and adjust drug dosage or select alternative psychiatric drugs less susceptible to microbial metabolism in these patients. Subsequently, monitoring biomarkers by tracking microbial metabolites or drug plasma levels in the patients to detect microbiome-mediated changes in drug activity. Furthermore, animal studies and limited clinical trials suggest fecal microbiota transplantation (FMT), of stool from a healthy donor into a patient’s gastrointestinal tract to restore microbial balance, can normalize gut microbiota, reduce systemic inflammation, and potentially improve behavioral or mood symptoms. Moreover, using probiotics and prebiotics to enhance the anti-inflammatory bacteria and reduce systemic and neuroinflammation while potentially improving the pharmacokinetics of psychiatric drugs by reducing microbial metabolism or sequestration. Clinical trials in depression and anxiety show improved mood and reduced inflammatory markers with probiotic supplementation, while prebiotics can enhance the growth of *Faecalibacterium* and *Coprococcus*, key SCFA producers associated with better drug efficacy.^10^ However, despite such therapeutic potential from these methods, they face important limitations, including the microbiome composition variability in all individuals, lack of standardized probiotic formulations in the market, and the safety concerns surrounding FMT. Thus, due to the experimental nature of these methods, considerable barriers must be overcome before these methods can be implemented in humans outside clinical trials.

## Conclusion

 The gut microbiome has a significant impact on health as well as neuropsychiatric disorders and psychiatric medications. An imbalance of the gut microbiome affects metabolic processes, thereby influencing neurotransmitter functions, and inflammation caused by neuropsychiatric disorders. The treatments such as fecal microbiome transplantation (FMT), application of probiotics, and prebiotics is premature and are investigational only. Hence, we would like to state that still elaborate researches are required to further link the strong association between the gut microbiome and its efficacy of neuropsychiatric drugs.

## Competing Interests

 The authors declare that there are no conflicts of interest.

## Ethical Approval

 Not applicable.
